# Vector-borne pathogens in cats and associated fleas in southern Ethiopia

**DOI:** 10.1186/s13071-025-06855-3

**Published:** 2025-06-19

**Authors:** Marika Grillini, Hana Tadesse, Alessandra Mondin, Maria Luisa Menandro, Giovanni Franzo, Giorgia Dotto, Antonio Frangipane di Regalbono, Bersissa Kumsa, Rudi Cassini, Giulia Simonato

**Affiliations:** 1 Department of Animal Medicine, Production and Health, University of Padova, 35020 Legnaro, PD Italy; 2 Arba Minch Agricultural Research Center, Southern Agricultural Research Institute, P.O. Box 2228, Arba Minch, Ethiopia; 3 Department of Veterinary Parasitology and Pathology, Addis Ababa University, P.O. Box 34, Bishoftu, Ethiopia

**Keywords:** Vector-borne pathogen, Cat, *Ctenocephalides felis*, *Echidnophaga gallinacea*, Ethiopia

## Abstract

**Background:**

The worldwide increment of cat populations has increased the risk of ectoparasite infestation and feline vector-borne pathogen (VBP) transmission. In low-income countries, such as Ethiopia, favorable climatic conditions and the absence of preventive measures against ectoparasites contribute to broadening VBP circulation. This study aimed to investigate the prevalence of protozoal (i.e., *Hepatozoon*, *Babesia*, and *Cytauxzoon* species) and bacterial (i.e., *Anaplasma*, *Ehrlichia*, *Rickettsia*, and *Bartonella* species) infections in owned cats and in their ectoparasites in southern Ethiopia.

**Methods:**

The study was conducted in four districts of the Gamo zone, southern Ethiopia. Cats were sampled, and information about the animals was recorded. Blood samples were collected on Flinders Technology Associates (FTA) cards, while ectoparasites were collected by combing and stored in 70% ethanol. Fleas were morphologically identified, and DNA was extracted from both blood samples and ectoparasites, then submitted to molecular analysis. Real-time polymerase chain reaction (PCR) and end-point PCR were used to detect pathogens. Positive samples were sequenced, and a phylogenetic analysis was performed on the obtained *Hepatozoon* spp. and *Rickettsia* spp. sequences.

**Results:**

Overall, 109 cats were sampled, and 115 fleas (i.e., 28 *Ctenocephalides felis* and 87 *Echidnophaga gallinacea*) and three ticks (*Haemaphysalis laechi*) were collected. Molecular analysis of feline blood samples revealed *Hepatozoon* spp. as the most common pathogen (36.7%; CI:28.3–46.1%), followed by *Rickettsia* spp. (5.5%; CI: 2.5–11.5%), *Bartonella* spp. (2.8%; CI:0.9–7.8%), and *Babesia* spp. (0.9%; CI:0.2–5.0%); whereas fleas harbored mostly *Rickettsia* spp. (52.2%; CI:43.1–61.1%), followed by *Bartonella* spp. (6.1%; CI: 3.0–12.0%), and *Hepatozoon* spp. (0.9%; CI: 0.2–4.8%). According to phylogenetic clustering, specimens of the *Hepatozoon* genus were classified as *H. felis*, *H. luiperdjie,*, and *H. canis*. Concerning the genus *Rickettsia*, it was not possible to reach a clear identification for the majority of the sequences, apart from some specimens ascribable to *R. felis* and *R. asembonensis*.

**Conclusions:**

Vector-borne pathogens posing significant threats to animal and human health were detected in this study. Molecular analysis suggested the circulation of different and genetically variable species in the feline host. The molecular approach allowed the identification of VBPs in the cat population and their fleas, providing new data on their occurrence and prevalence in Ethiopia and, more generally, in sub-Saharan Africa.

**Graphical abstract:**

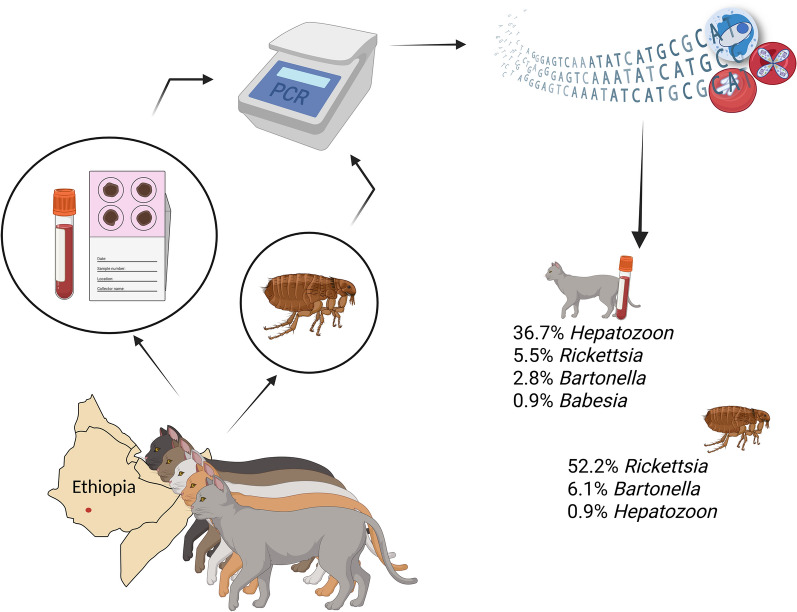

**Supplementary Information:**

The online version contains supplementary material available at 10.1186/s13071-025-06855-3.

## Background

Climate change and the increased mobility of people and their pet animals have contributed to the spread of vectors and pathogens, leading to an increasing risk of transmission of diseases carried by these vectors, in particular for companion animals [1]. In addition, the human-induced environmental alterations (i.e., expansion of farmland, urbanization, deforestation, and establishment of settlements in natural ecosystems) promote the risk of spill-over between wild and domestic animals, as well as the opportunities for novel disease transmission cycles to develop [2]. These trends also concern cats, which evolved from “hunting” animals (i.e., mice catchers) to social creatures who provide companionship for humans, becoming common pets worldwide [3], with increased cohabitation with their owners in urban and rural environments [4].

The main ectoparasites that can be detected in cats are fleas and ticks, although the prevalence of the latter is generally lower than that reported in dogs. Indeed, among vector-borne pathogens (VBPs), tick-borne pathogens (TBPs) are mostly found in dogs, whereas flea-borne pathogens are found in cats [5]. Concerning fleas, *Ctenocephalides felis* is the most relevant, along with *C. canis* and *Pulex irritans*, which can rarely be found on cats [6]. Moreover, *Echidnophaga gallinacea,*, the sticktight flea, is known to be commonly found on peridomestic avians, but it can occasionally infest domestic felids, rodents, horses, and even humans, mostly in warm areas of the world [6–11]. Conversely, different ticks have been described to infest cats according to the geographical area (*Ixodes* and *Dermacentor* species mostly in the northern hemisphere, *Amblyomma* and *Haemaphysalis* mostly in Africa and Asia, and *Rhipicephalus* worldwide [7, 12–16]).

Many VBPs can infect felines, including flea-associated pathogens belonging to the genera *Bartonella* (e.g., *Bartonella henselae* and *Bartonella clarridgeiae*) and *Rickettsia*, along with many TBP species (i.e., *Hepatozoon* spp., *Babesia* spp., *Cytauxzoon* spp., *Anaplasma* spp., *Ehrlichia* spp., *Rickettsia* spp., and *Borrelia* spp.) [5, 17]. Cats are the main mammalian reservoir of *B. henselae* and *B. clarridgeiae*, two species that have been associated with cat scratch disease in humans [18–20], and *C. felis* is considered the main vector. Though *Rickettsia felis*, the zoonotic agent of the flea-borne spotted fever [21, 22], is mainly transmitted by *C. felis*, it has also been detected in other arthropod taxa [23]. Conversely, different genera of ticks (such as *Amblyomma*, *Dermacentor*, *Haemaphysalis*, *Ixodes,*, and *Rhipicephalus*) are probably involved in the transmission of feline TBPs [24], although the epidemiological information available on these latter genera is still limited. Among others, feline hepatozoonosis’ etiological, epidemiological, and clinical features are still mostly unknown worldwide. Five species (i.e., *Hepatozoon felis*, *H. silvestris*, *H. canis*, *H. luiperdjie,*, and *H. ingwe*) have been reported in wild and domestic felids in several countries [25–27], but their taxonomic classification is still evolving. Hepatozoonosis is often asymptomatic [28], with few clinical cases reported and described [29, 30], and ticks seem to be the most likely arthropod vectors [31].

In low-income countries with a tropical climate, such as Ethiopia, the presence of favorable weather conditions, poor general hygiene, and the lack of prevention and control measures make arthropods very common and widely distributed in pet animals [32]. In the absence of adequate treatment against ectoparasites, these animals can act as vectors and transmit pathogens to pets that in turn could act as carriers or as competent reservoirs [33]. Nevertheless, their role in the epidemiology of VBPs in sub-Saharan Africa has not been adequately investigated [2, 34].

In Ethiopia, the research on the epidemiology of feline VBPs has been limited to a few studies. Five species of flea (i.e., *C. felis*, *C. canis*, *P. irritans*, *E. gallinacea,*, and one single occasion of infestation with *Xenopsylla cheopis*), and ticks of the genera *Amblyomma*, *Haemaphysalis*, and *Rhipicephalus* have been reported in Ethiopian cats [7, 12]. Concerning the pathogens, *R. felis* and *B. henselae* have been recently detected molecularly in cat fleas [32]. Thus, the purpose of the present study was to investigate the occurrence and prevalence of protozoal (i.e., *Hepatozoon* , *Cytauxzoon*, and *Babesia* species) and bacterial (i.e., *Anaplasma*, *Ehrlichia*, *Bartonella*, and *Rickettsia* species) pathogens using molecular methods in owned cats and associated ectoparasites in selected areas of southern Ethiopia.

## Methods

### Study area and sampling

The Gamo Zone is located in the Southern Nations, Nationalities, and People Region, between 5°55′ N and 6°20′ N latitude and between 37°10′ E and 37°40′ E longitude. The survey was performed between November 2020 and January 2021 in four districts of the Gamo Zone in southern Ethiopia, i.e., Arba Minch town, Chencha town, Arba Minch Zuria, and Gerese. Only clinically healthy cats were recruited, whose owners verbally consented to participate in the present study.

Information on sex (i.e., male, female), age classes (i.e., young: 0–6 months, adult: > 6 months), location (i.e., urban, rural), lifestyle (i.e., indoor, mixed indoor–outdoor, outdoor), agroecology (i.e., highland, midland, lowland) and the presence of ectoparasites was recorded for each cat.

Overall, 109 domestic cats were recruited and investigated through a clinical examination by the veterinarian before the blood sampling. Together with a scrupulous observation, the fur was brushed using a standard fine metal flea comb (12 teeth per 1 cm) for 10–15 min [12, 35] to look for possible ectoparasites. After combing, the comb was held over a white plastic tray and ectoparasites were collected from there. The isolated ectoparasites (115 adult fleas and 3 ticks) were moved into labeled specimen bottles containing 70% ethanol.

Cats’ whole blood was collected from the cephalic vein, and 100 μL was adsorbed on the classic Flinders Technology Associates (FTA™) Nucleic Acid Collection Cards (Whatman^®^, Maidstone, UK). Subsequently, FTA cards were dried, coded with sequential numbers, and stored in sealed FTA pouches with a silica gel desiccant (Sigma Aldrich, Co., Life Sciences, St. Louis, MO, USA) until analysis.

The study protocol was approved by the Animal Ethics Committee of Addis Ababa University, College of Veterinary Medicine and Agriculture (agreement no. VM/ERC/01/13/021).

### Ectoparasite identification, molecular analysis, and sequencing

Ectoparasites were counted and morphologically identified at the species level, determining the sex, at the Laboratory of Parasitology of the Addis Ababa University, College of Veterinary Medicine and Agriculture, Bishofu, Ethiopia, using a stereo microscope and according to identification keys [36, 37].

Thereafter, FTA cards and ectoparasites were transferred to the Laboratory of Parasitology of the University of Padua, Legnaro (PD), Italy, as per authorization of the Italian Ministry of Health (Prot. N. 0002711, 22 April 2021), to perform molecular analysis. DNA was extracted from dried blood samples in each FTA card and from each ectoparasite individually, according to the manufacturer’s instructions using the NucleoSpin™ Tissue extraction kit (Macherey–Nagel, Düren, Germany) and DNeasy Blood and Tissue kit (Qiagen, Hilden, Germany), respectively. All DNA samples were stored at −20 °C until molecular analysis execution.

A real-time PCR was run using QuantiNova SYBR^®^ Green PCR Kit (QIAGEN Group, Hilden, Germany) with primers previously described by Tabar et al. [38] (Table 1), targeting the *18S-*rRNA gene, spanning the V4 region, to detect protozoal pathogens (i.e., *Babesia* spp., *Cytauxzoon* spp., and *Hepatozoon* spp.). The assay was performed in the Roche LightCycler^®^ 96 thermocycler (La Roche Ltd, Basel, Switzerland) according to the following thermal protocol: incubation at 95 °C for 2 min, followed by 45 cycles of amplification steps at 95 °C for 5 s and 60 °C for 10 s, concluding at 95 °C for 10 s. Melting curve analysis was performed by increasing the temperature (ramp rate 0.2 °C/s) from 65 °C to 97 °C and continuously monitoring the fluorescence data. The specificity of the obtained amplicons was evaluated through the melting temperature curve analysis as previously described [39]. Then, positive samples were submitted to an end-point PCR with the same primers and sequenced as described below. Furthermore, whenever possible, selected positive samples were analyzed by a second end-point PCR, using a couple of primers previously described by Bhoora et al. [40] targeting the *18S-*rRNA gene, in order to obtain a longer amplicon to confirm the species with higher confidence (Table 1).

**Table 1:** Primers used to target different vector-borne pathogens and the expected amplicon lengths

Pathogen	Primers	Gene/amplicon length	Annealing temperature	References
Apicomplexa (*Babesia* spp., *Cytauxzoon* spp., *Hepatozoon* spp.)	Piro A CCAGCAGCCGCGGTAATTC Piro B CTTTCGCAGTAGTTYGTCTTTAACAAATCT	*18S* rRNA/373 bp	TD 60 → 64 °C (−0.5 °C/cycle)	[38]
Apicomplexa (*Babesia* spp., *Cytauxzoon* spp., *Hepatozoon* spp.)	NBabesia1F AAGCCATGCATGTCTAAGTATAAGCTTTT BT18S3R CCTCTGACAGTTAAATACGAATGCCC	*18S* rRNA/800 bp	58° C	[40]
*Bartonella* spp.	BarF1 CTCTTTCTTCAGATGATGATCC Bar R1 AACCAACTGAGCTACAAGCCCT	*ITS*/145–251 bp	TD: 62 → 58 °C (−0.5 °C/cycle)	[41]
*Anaplasma* spp./*Ehrlichia* spp.	PER1 TTTATCGCTATTAGATGAGCCTATG PER2 CTCTACACTAGGAATTCCGCTAT	*16S* rRNA/451 bp	TD: 60 → 45 °C (−0.5 °C/cycle)	[42]
*Anaplasma phagocytophilum*/*Anaplasma platys*	Ephpl-569F ATGGTATGCAGTTTGATCGC Ephpl-1193R TCTACTCTGTCTTTGCGTTC	*groEL*/624 bp	59 °C	[83]
*Rickettsia* spp.	Rp877p GGGGGCCTGCTCACGGCGG Rp1258n ATTGCAAAAAGTACAGTGAACA	*gltA*/381 bp	TD: 60 → 54 °C (−0.5 °C/cycle) TD: 58 → 54 °C (−0.5 °C/cycle)	[84] [44]
	Rp896p GGCTAATGAAGCAGTGGATAA Rp1233n GCGACGGTATACCCATAGC	*gltA* nes/338 bp		
	Rc.rompB.4362p GTCAGCGTTACTTCTTCGATGC Rc.rompB.4836n CCGTACTCCATCTTAGCATCAG Rc.rompB.4496p CCAATGGCAGGACTTAGCTACT Rc.rompB.4762n AGGCTGGCTGATACACGGAGTAA	*ompB/*475 bp *ompB* nes*/*276 bp	TD: 60 → 54 °C (−0.5 °C/cycle) TD58 → 54 °C(−0.5 °C/cycle))	[45]
	RompB OF GTAACCGGAAGTAATCGTTTCGTAA RompB OR GCTTTATAACCAGCTAAACCACC RompB SFG IF GTTTAATACGTGCTGCTAACCAA RompB SFG/TG IR GGTTTGGCCCATATACCATAAG RompB TG IF AAGATCCTTCTGATGTTGCAACA	*ompB/*512 bp *ompB* nes*/*426 bp 250 bp	TD: 59 → 54 °C (−0.5 °C/cycle) 56 °C 56 °C	[44]
	17KDa5 GCTTTACAAAATTCTAAAAACCATATA 17KDa3 TGTCTATCAATTCACAACTTGCC 17KDa1 GCTCTTGCAACTTCTATGTT 17KDa2 CATTGTTCGTCAGGTTGGCA	*htrA/*549 bp *htrA* nes*/*434 bp	58 °C TD58 → 48*C (−0.5 °C/cycle)	[46] [47]
Fleas (Siphonaptera)	F-Leu TCTAATATGGCAGATTAGTGC R-Lys GAGACCAGTACTTGCTTTCAGTCATC	*COII*/780 bp	TD: 57 → 47 °C (−0.5 °C/cycle)	[48]

TD, touch-down

The presence of bacterial pathogens was assessed using specific end-point PCR assays. *Bartonella* spp. DNA was detected by targeting the rRNA *16S*–*23S* intergenic sequences (*ITS*), as described by Jensen and colleagues [41]. *Anaplasma* spp. and *Ehrlichia* spp. were screened using a protocol amplifying a portion of the *16S-*rRNA gene [42]. *Anaplasma phagocytophilum*, *A. platys,*, and *Rickettsia* spp. were detected using specific end-point PCR assays amplifying portions of the *A. phagocytophilus groEL*, *A. platys groEL*, and *Rickettsia gltA* encoding genes [43]. Whenever possible, to confirm *Rickettsia* identification, *gltA*-positive samples underwent further analysis using nested end-point PCR assays targeting portions of the *ompB* and *htrA* genes using primers already described [44–47]. All end-point PCR reactions were performed using a Biometra T Advanced thermocycler (Analytic Jena, Gottingen, Germany), Phire Hot Start II PCR Master Mix (Thermo Scientific, Vilnius, Lithuania), and 0.3 μM of each primer. Primer sequences, expected product length, and annealing temperature have been summarized in Table 1. Each PCR reaction included at least one positive and one negative control. Amplification products were visualized by electrophoresis on 2% agarose gels stained with SybrSafe DNA Stain (Invitrogen, Thermo Scientific, Carlsbad, CA, USA).

For flea samples, initial screening analyses were performed on pools containing DNA of up to three individual ectoparasites of the same origin, species, and sex. DNA from positive pools was subsequently analyzed individually to identify the positive samples.

Amplicons were purified using EXOSAP-it^®^ (ExoSAP-IT™ Express PCR Product Cleanup Reagent, Thermo Fisher Scientific Inc., Waltham, MA, USA) according to the manufacturer’s instructions. Purified products were then submitted to bidirectional Sanger sequencing (Macrogen Spain, Madrid, ES, and Macrogen Italy, Milan, IT), using the same primers used in the PCR assays. Nucleotide sequences were edited using ChromasPro v.2.1.8 (Technelysium Pty Ltd., Brisbane, Australia) and compared among themselves and to those in GenBank^®^ using the ClustalW alignment tool (http://npsa-prabi.ibcp.fr) and BLAST software (https://blast.ncbi.nlm.nih.gov/Blast), respectively. All sequences were deposited in GenBank and used for the successive phylogenetic analysis. Rickettsial-specific identification was considered, confirmed, and reported in the text only when BLAST analysis results consistently showed the highest percentage of similarity to a single species across at least two different genes among those tested (*gltA* and *ompB* and/or *htrA*).

Finally, to confirm morphological identification of fleas, an end-point PCR amplifying a 780 bp long portion of the flea cytochrome oxidase II (*COII*) gene was performed on DNA samples extracted from fleas, using the F-Leu and R-Lys primer pair [48]. The resulting amplicons were purified and then subjected to Sanger sequencing.

### Data analysis

Prevalence values and their 95% confidence intervals (95% CI) were calculated through EpiTools (www.epitools.ausvet.com.au) using the Wilson method for each identified pathogen [49]. Sex, age, location, lifestyle, agroecology, and presence of fleas were used as variables to calculate proportions for all pathogen species sufficiently prevalent and summarized descriptively. The Pearson Chi-squared test, or Fisher’s exact test when appropriate, was used to compare proportions for all identified pathogens with convenient prevalence values, using IBM SPSS Statistics, version 28. A significance level of *P* < 0.05 was considered statistically significant.

### Phylogenetic analysis

Phylogenetic analyses were conducted for the genera *Hepatozoon* and *Rickettsia* separately. The *Hepatozoon 18S* rRNA sequences and the rickettsial *gltA* gene sequences obtained from positive cat blood samples and fleas were aligned with a carefully curated set of reference sequences. The rickettsial reference sequences were selected to comprehensively represent species belonging to the spotted fever group, the typhus group, and the transitional group. The multiple sequence alignment was performed using MAFFT [50] with default parameters, and a maximum likelihood phylogenetic tree was reconstructed on the basis of the obtained alignment using IQ-Tree [51] for each genus, to evaluate the clustering of detected strains with reference ones, and to identify the taxonomic relationships. The best substitution model was selected on the basis of the Bayesian information criterion (BIC) calculated using the same program. The branch support was assessed by performing 10,000 bootstrap replicates.

## Results

### Feline population description and ectoparasite identification

Out of the 109 domestic cats sampled, the majority (*n* = 40; 36.7%) came from Arba Minch town, followed by Arba Minch Zuria district (*n* = 32; 29.4%), Gerese district (*n* = 31; 28.4%), and Chencha town (*n* = 6; 5.5%). Overall, 66 cats (60.6%) were female, and 43 were male (39.4%). The cat population ranged from 1 to 72 months of age. Individual data of cats are reported in Table 2. The detailed distribution of sampled animals in each district can be found in Additional file 1: Table S1.

**Table 2:** Distribution of the cat population (*n* = 109) according to the considered factors

Animal data		Number of cats (%)
Sex	Male	43 (39.4%)
	Female	66 (60.6%)
Age class	Adult (> 6 months)	83 (76.1%)
	Young (≤ 6 months)	26 (23.9%)
Lifestyle	Indoor	89 (81.7%)
	Mixed	16 (14.7%)
	Outdoor	4 (3.7%)
Agroecology	Lowland	51 (46.8%)
	Midland	21 (19.3%)
	Highland	37 (33.9%)
Flea infestation	Present	24 (22.0%)
	Absent	85 (78.0%)

The 115 adult fleas were identified morphologically as 28 *C. felis* (males = 7; females = 21) and 87 *E. gallinacea* (males = 12; females = 75), and molecularly confirmed in a portion of the specimens (5 for each species). Their distribution in the investigated cat populations has already been reported in a previous publication [52] and compared with ectoparasite patterns in sympatric dog populations. In addition, three *Haemaphysalis laechi* female ticks were isolated from three different cats.

### Molecular analysis in cats

Overall, 42.2% (46/109) of the examined cats showed molecular positivity to at least one of the investigated pathogens. Nearly one-third of the tested blood samples were positive for *H. felis.*. In addition, a small number of animals tested positive for other pathogens, including *H. luiperdjie*, *B. clarridgeiae*, *B. henselae*, *R. felis*, *Rickettsia* sp., *H. canis*, and *Babesia leo*, as shown in Table 3. The GenBank accession numbers of the respective sequences are provided in Additional file 2: Table S2.

**Table 3:** Confirmed protozoal and bacterial pathogens isolated from the blood of the investigated cat population (*n* = 109)

Pathogen		Number of positive cats (%; 95% CI)
Protozoal pathogens	*Hepatozoon felis*	33 (30.3; 22.4–39.5)
	*Hepatozoon luiperdjie*	6 (5.5; 2.6–11.5)
	*Hepatozoon canis*	1 (0.9; 0.2–5.0)
	*Babesia leo*	1 (0.9; 0.2–5.0)
Bacterial pathogens	*Bartonella clarridgeiae*	2 (1.8; 0.5–6.4)
	*Bartonella henselae*	1 (0.9; 0.2–5.0)
	*Rickettsia felis*	1 (0.9; 0.2–5.0)
	*Rickettsia* spp.	5 (4.6; 2.0–10.3)

In total, 33 *Hepatozoon* positive samples revealed a high percentage of identity (99–100%) with *H. felis*, and 6 samples were identified as *H. luiperdjie* (percentage of identity 99.6–100%) at BLAST analysis. Sequences with higher identity in GenBank^®^ were isolated in domestic cats in Angola [53] and in southwestern Africa [54]. One sample showed 100% sequence identity with *H. canis* [55] from caracals (*Caracal caracal*) in South Africa. Finally, the *B. leo* identified in one cat showed a 100% identity to those isolated from domestic cats in South Africa [56]. Two positive samples attributable to *H. luiperdjie* and the unique *B. leo* were further confirmed by the second end-point PCR, obtaining a longer fragment, again showing a high percentage of identity (99–100%) with sequences from domestic cats of South Africa [54, 56]. Due to insufficient material, it was impossible to process the other four samples positive for *H. luiperdjie*.

Regarding bacterial pathogens, successful amplification of the *Bartonella* spp. *ITS* region and rickettsial *gltA* gene portion were achieved from 3 (2.8%) and 6 (5.5%) of the 109 cat blood samples, respectively. BLAST analysis of the three *Bartonella* nucleotide sequences revealed that two of them were identical to *B. clarridgeiae* and one was highly similar to *B. henselae* (99.2%). The nucleotide *gltA* sequences confirmed the *Rickettsia* genus for all six positive samples, and one of them showed a high percentage of identity with the sequences of *R. felis* deposited in Genbank for both *gltA* and *ompB* (100% identity). This sample was collected from a young female cat living in Arba Minch town. No positive results were recorded for *Cytauxzoon* spp., *Anaplasma* spp., and *Ehrlichia* spp.

Statistical analysis was performed only for *H. felis* (30.3% of overall prevalence), since all other pathogens in cats showed low prevalence levels. The agroecology was the only factor significantly associated with positivity for *H. felis* (*P* = 0.002), with an increasing trend in prevalence values from lowland (15.7%), to midland (26.6%), to highland (51.4%).

### Molecular analysis in fleas

On the basis of species, sex, and provenance, fleas were grouped into 41 pools (11 pools of *C. felis* and 30 of *E. gallinacea*). The comparison between positivity rates in the two flea species for all encountered pathogens is reported in Table 4.

**Table 4:** Positivity rate of the different pathogen species identified in the two flea species isolated from the investigated cat population

Pathogen		*Ctenocephalides felis* *n* = 28	*Echidnophaga gallinacea* *n* = 87
Genus	Species	No. positive (%; 95% CI)	No. positive (%; 95% CI)
*Hepatozoon*	*H. felis*	0 (0; 0–12.1)	1 (1.1; 0.2–6.2)
*Bartonella*	*B. clarridgeiae*	0 (0; 0–12.1)	6 (6.9; 3.2–14.2)
	*B. elizabethae*	0 (0; 0–12.1)	1 (1.1; 0.2–6.2)
*Rickettsia*	*R. asembonensis*	20 (71.4; 52.9–84.7)	1 (1.1; 0.2–6.2)
	*R. felis*	1 (3.6; 0.6–17.7)	0 (0; 0–4.2)
	*Rickettsia* spp.	2 (7.1; 2.0–22.6)	36 (41.4; 31.6–51.9)

Only one pool of three females of *E. gallinacea* from Arba Minch town tested positive for *Hepatozoon* spp. After individual analysis, one out of three was confirmed positive with 99% of sequence identity compared with *H. felis* reported in domestic cats in Luanda, Angola [48] (GenBank: MG386482.1).

Five flea pools and subsequently eight individual fleas tested positive for *Bartonella* species. BLAST analysis revealed that the only sequence obtained from *C. felis* was identical to the uncultured *B. clarridgeiae* clone MU9/KCF21 (GenBank: HM990962), while within the seven detected in *E. gallinacea,*, six were identical or highly similar (98.6–100%) to *B. clarridgeiae*, and one was identical to *Bartonella elizabethae* (GenBank: LR134527).

DNA of *Rickettsia* spp. was detected in 9 pools of *C. felis* and 19 of *E. gallinacea* by amplification of the *gltA* gene. Subsequent individual analysis revealed 60 positive fleas for *Rickettsia* spp., with a percentage of identity of *gltA* gene portion sequences ranging from 99 to 100% compared with those reported in Genbank. Species classification was assigned to 22 samples, for which BLAST analyses yielded consistent results across at least two different genes: 21 closely matched with *Rickettsia asembonensis* and 1 with *R. felis* (Table 4).

### Phylogenetic analysis

**Fig. 1:**
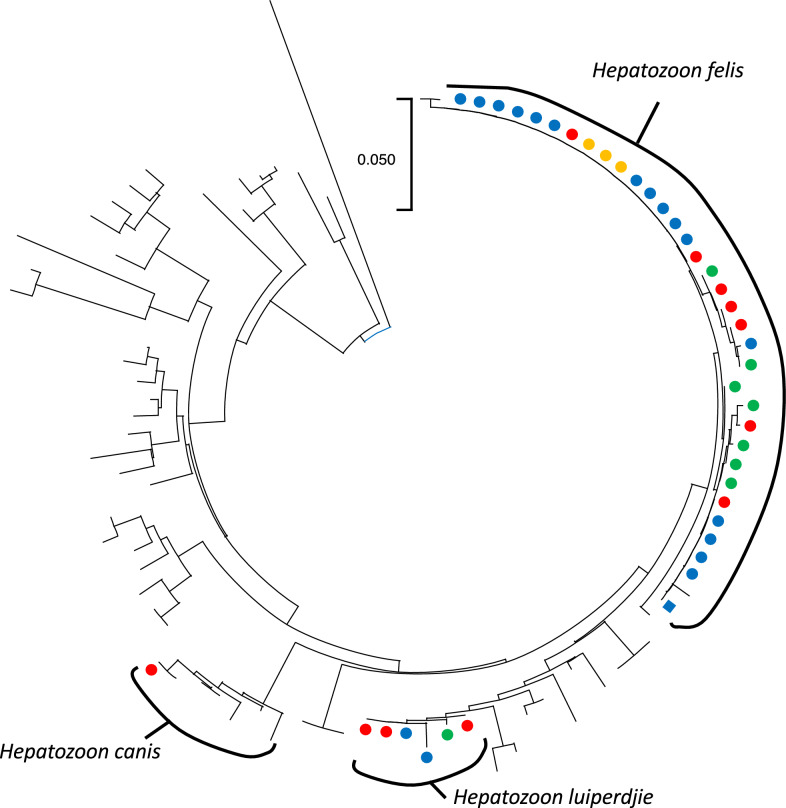
Maximum likelihood phylogenetic tree of the obtained *Hepatozoon* sequences plus a set of reference ones. Sequences obtained from cats have been marked with a filled circle, while the one obtained from *Echidnophaga gallinacea* has been marked with a filled square. Collection areas have been color-coded: Arba Minch town in green, Gerese district in blue, Arba Minch Zuria district in red, and Chencha town in yellow. Further details, sequence name, and bootstrap support are available in Additional file 3: Fig. S1

Because of the detected genetic variability within the samples that were positive for *Hepatozoon* spp. and *Rickettsia* spp., two phylogenetic analyses were performed for these groups of pathogens. Concerning *Hepatozoon* spp., two clear clusters were identified (Fig. 1; Additional file 3: Fig. S1), corresponding to *H. luiperdjie* and *H. felis.*. Some sub-clusters could be identified within the *H. felis* sequences obtained in the present study, although not always featured by a high bootstrap support. Both *H. luiperdjie* and *H. felis*, and different sub-clades of *H. felis*, included strains originating from different regions. The only sequence obtained from fleas (i.e., from *E. gallinacea*) was identical to others collected from domestic cats, including one strain from the same region.

**Fig. 2:**
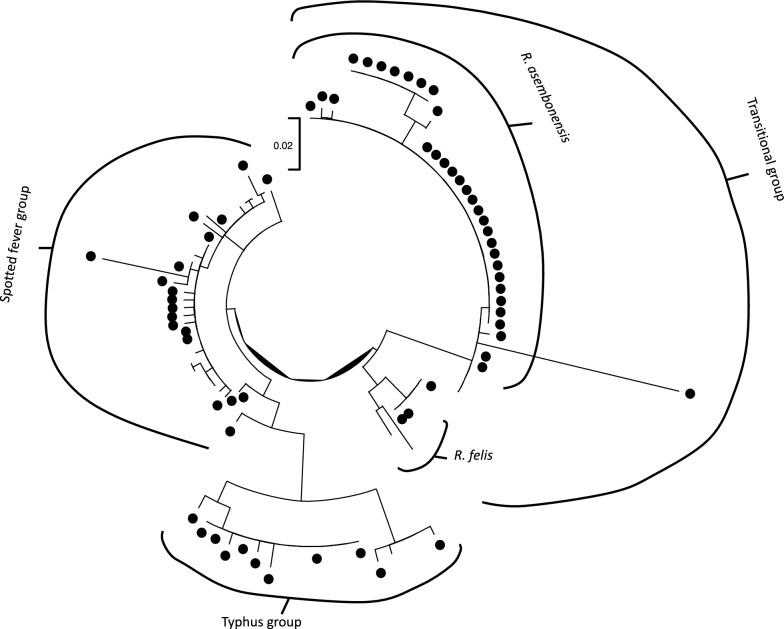
Maximum likelihood phylogenetic tree of the obtained *Rickettsia* sequences plus a set of reference ones. Sequences obtained in this study have been marked with a filled black circle. Further details, sequence name, and bootstrap support are available in Additional file 4: Fig. S2

Regarding *Rickettsia* spp., the phylogenetic tree obtained from the *gltA* gene sequences highlights their clustering, with high bootstrap support, into the three groups: the transitional group, the spotted fever group, and the typhus group (Fig. 2; Additional file 4: Fig. S2). However, a limited number of strains (i.e., four sequences) were distantly related to the selected references, hampering their classification with confidence. For most of the sequences falling within the transitional group, further analyses performed by amplifying the *ompB* and *htrA* genes yielded sequences with a high percentage of homology to *R. asembonensis* and *R. felis* (Tables 3 and 4).

## Discussion

The present study provides a comprehensive picture of the presence and distribution of VBPs within the considered cat population and their associated fleas. The molecular approach employed herein allowed the identification of many pathogens relevant for animal health and four species known to have zoonotic potential, providing valuable insights on their occurrence and prevalence in Ethiopia and, more generally, in sub-Saharan Africa. We opted for the sole molecular approach, which can be easily performed on blood samples preserved in FTA cards, also in consideration of the difficulties for inexperienced staff to reach specific identification in stained blood smears.

The ectoparasites mostly associated with the investigated cat population were flea species. *Ctenocephalides felis* showed a higher prevalence, but at low infestation burdens, while *E. gallinacea* presented a higher average number of individuals per cat. Worldwide, *C. felis* is the foremost ubiquitous flea species parasitizing pets [57], indeed, several studies reported a high percentage (up to 72%) of infested animals among free-roaming cats [58]. Conversely, the high burden of the sticktight flea in a few cats found in our study may be due to a temporary infestation resulting from the close contact between free-ranging cats and infested birds, demonstrating the adaptability of *E. gallinacea* in terms of its choice of hosts [8]. Although little information was available regarding the Ethiopian scenario, Kumsa et al. [7] stated that both species are found in domestic cats in central Ethiopia, as confirmed by our findings. On the contrary, only three *H. laechi* ticks were isolated from three cats, supporting the concept that cats are almost free from ticks, when compared with dogs, probably due to a lower susceptibility linked to self-grooming [5]. Hence, ticks were not included in the molecular analysis, since the low number would not have allowed any significant inference.

In the cat population investigated in this study, *Hepatozoon* species proved to be the most frequent protozoan parasites, while the detection of *Babesia* species was rarer. The *Hepatozoon* species most prevalent in the studied cats was *H. felis*, with a prevalence value (30%) comparable to what was reported in other populations [59, 60]. On the basis of the phylogenetic clustering, most of the detected strains were classified as *H. felis*, although a certain genetic variability was observed within the obtained sequences. This is in line with the literature, since some authors suggested using the definition of *H. felis* complex, considering the genetic differences among isolates attributed to this species in different studies [27]. Interestingly, no geographical clustering was apparent in our isolates, suggesting a quite broad circulation and strain interchange between the different districts of the study area. The strain identified from *E. gallinacea* was identical to that identified from cats of the same area. The role of *E. gallinacea* in *Hepatozoon* transmission remains to be further investigated, also considering the high number of fleas of this species encountered in this cat population. However, the positivity may be the result of recently consumed infected blood rather than the flea itself acting as a potential biological vector. Furthermore, other transmission routes could be considered, such as vertical transmission or the predation of a paratenic host by cats (e.g., rodents), as already demonstrated for other *Hepatozoon* species [25].

Although *H. felis* is the main *Hepatozoon* species occurring in felines, other species were also reported, such as *H. canis* [61], which is, despite the name, also sporadically detected in cats [61–63], probably as a consequence of low host specificity of the protozoa [28] and of the two host species living frequently in sympatry. Therefore, the finding of a sample with an infection probably attributable to *H. canis* is not surprising, since this pathogen can be accidentally found in cat blood, considering the previously assessed circulation of this pathogen among dogs from the same areas [43].

The epidemiology of the other two species found in the investigated cats (i.e., *H. luiperdjie* and *B. leo*) is still poorly studied, and the mammal species acting as the main reservoir have not yet been identified. Interestingly, to the best of the authors’ knowledge, this is the first report for both species so far in Ethiopia. Specifically, very scant data are available about the epidemiology of *H. luiperdjie* infection, since it has been recently reported only in wild felids in South Africa [27, 54]. Of note, the *H. luiperdjie* positive cats herein identified were geographically distant, potentially suggesting an involvement of other reservoir hosts, rather than cats (e.g., wild mammals), in the life cycle perpetuation. While *B. leo* infection in domestic cats may clinically develop with lethargy, icterus, and pyrexia [56], the infected cat here identified did not show any detectable sign of acute/severe *Babesia* infection, advocating for future studies to better elucidate the *B. leo* infection pathogenesis.

Finally, the finding of no cats positive for *Cytauxzoon* spp. is consistent with previous data from South Africa, and this may be explained by the absence of established populations of wild felid species, which are known to act as reservoirs in other geographical contexts [64].

Regarding bacterial pathogens, two species of *Bartonella* (*B. henselae* and *B. clarridgeiae*) were identified in cat blood samples, and two species (*B. clarridgeiae* and *B. elizabethae*) were identified in fleas. The presence of zoonotic agents, such as *B. henselae*, *B. clarridgeiae,*, and *B. elizabethae,* is noteworthy, although *Bartonella* spp. circulation was already reported in the same hosts in Africa [32, 34, 65–67], and both *B. clarridgeiae* and *B. henselae* have already been detected in Ethiopia. In the present study, the positivity for *B. henselae* and *B. clarridgeiae* was low in both cats and *C. felis*, while a higher positivity rate for *B. clarridgeiae* was detected in *E. gallinacea*. *Echidnophaga gallinacea* has rarely been associated with *Bartonella* spp. [34, 68], but its role in the cycle and route of transmission of *Bartonella* needs to be better investigated; indeed, Madder and colleagues [34] reported that *Bartonella* spp. are equally linked to *C. felis* and *Echidnophaga* spp. collected from cats. Furthermore, one specimen of *E. gallinacea* harbored *B. elizabethae*, which is usually associated with rats and their fleas [69], but it has also been detected in cats, dogs, and humans [65, 70, 71]. To date, the prevalence of *Bartonella* species reported in the literature is probably underestimated owing to the specificity of the technique used for the diagnosis, since only *B. henselae* detection or a generic molecular identification was implemented in former studies [32, 34]. A specific identification of circulating pathogens, as performed in this study, can contribute to clarify the epidemiology of these infections.

Rickettsial bacteria were the most commonly found in this study (*n* = 66; 6 blood samples from cats, 60 isolates from fleas) and the phylogenetic analysis (Additional file 4: Fig. S2) showed that most of the sequences obtained, amplifying a portion of the genus-specific *gltA* gene, clustered into the transitional group (*n* = 36), while fewer sequences clustered into the spotted fever group (*n* = 15) and the typhus group (*n* = 11). A few sequences (*n* = 4) were distantly related to the above-mentioned groups. For most of the sequences falling within the transitional group, further analyses performed by amplifying the *ompB* and *htrA* genes yielded sequences with a high percentage of homology to *R. asembonensis* (*n* = 21) and *R. felis* (*n* = 2).

Our study, therefore, confirms the circulation of *R. felis*, although at a low level [32]. Cats and dogs are the main hosts of this pathogen, but *R. felis* can infect several animals, and it is considered a common cause of fever in humans in Africa [72] and the occurrence of this species has already been reported in Ethiopia [32, 73, 74]. Furthermore, specimens of both *C. felis* (71.4%) and *E. gallinacea* (1.1%) were found with isolates highly similar to those of *R. asembonensis* for at least two different genes, with a significantly higher infection rate in the former. *Rickettsia asembonensis* is a *R. felis*-like organism detected and isolated for the first time in Kenya [75, 76]. Its pathogenicity is unclear, but it was identified in a patient with febrile symptomatology, myalgia, arthralgia, mild headache, and the presence of petechiae in Malaysia [77], and in patients with nonspecific acute febrile syndrome in Peru [78]. The close match with *R. asembonensis* in fleas of the study area confirmed what was reported in other studies [79–82] and, considering its absence in cats, calls for investigating other potential vertebrate mammals to identify their epidemiological roles.

For many samples that yielded positive results for the genus *Rickettsia*, it was not possible to achieve a specific identification. However, the distribution of many isolates within the phylogenetic tree in the spotted fever and typhus groups, which includes agents responsible for important zoonoses such as murine typhus (*Rickettsia typhi*) and epidemic typhus (*Rickettsia prowazekii*), calls for further studies, possibly based on longer DNA fragments.

## Conclusions

This study provided new molecular data, contributing to identifying the circulation of vector-borne bacteria and protozoa infections in domestic cats. Our findings highlighted a huge diversity of pathogens in cat and flea populations under investigation, providing new insights into their epidemiology, although the high genetic variability hampered the specific identification of many isolates. In addition, the study revealed the presence of zoonotic pathogens, such as *Bartonella* and *Rickettsia* species, indicating a possible widespread local circulation.

Further research is needed to understand the epidemiology of these pathogens and assess the risk of human infection. A more comprehensive approach, based on multiple genes and longer DNA fragments, is recommended for the more relevant pathogens detected in this area, starting from the zoonotic ones (e.g., *Rickettsia*). Notwithstanding, the limited economic resources are an important constraint in this specific context, the results of this study may stimulate more research efforts to manage potential health risks for both cats and humans in sub-Saharan Africa.

## Supplementary Information

Additional file 1: Table S1. Individual data of the cat population distributed in the four investigated sites

Additional file 2: Table S2. ID numbers of the sequences obtained from positive samples and deposited in GenBank

Additional file 3: Fig S1. Maximum likelihood phylogenetic tree of the obtained *Hepatozoon* sequences plus a set of reference ones. Bootstrap support values higher than 70% have been reported near the corresponding nodes. Sequences obtained from cats have been marked with a filled circle, while the one obtained from *Echidnophaga gallinacea* has been marked with a filled square. Collection areas have been color-coded: Arba Minch town in green, Gerese district in blue, Arba Minch Zuria district in red, and Chencha town in yellow

Additional file 4: Fig S2. Maximum likelihood phylogenetic tree of the obtained *Rickettsia* spp. *gltA* gene portion sequences obtained in the present study, plus a set of reference ones. Sequences obtained from cats have been marked with a filled circle. Branch support is reported near the corresponding node; only values higher than 70 have been maintained for clarity reasons. The use of boxes with colored background highlights the clustering within the spotted fever group (light yellow), typhus group (light blue), and transitional group (light green). Four sequences (i.e., PP886936, PP886913, PP886921, PP886935) are poorly related to the selected references and therefore are not included in the colored areas.

## Data Availability

Main data (e.g., sequence accession numbers) are provided within the manuscript or supplementary information files. Files containing the raw data supporting our findings can be requested directly from the corresponding author.

## Abbreviations

BIC: Bayesian information criterion
COII: Mitochondrial cytochrome oxidase II
FTA: Fast technology for analysis of nucleic acids
gltA: Citrate synthase
htrA: Stress response of the high-temperature requirement A family
ITS: Internal transcribed spacer
ompB: Outer membrane protein B
PCR: Polymerase chain reaction
VBP: Vector-borne pathogen
