# Supplementary material for: Vector-borne pathogens in cats and associated fleas in southern Ethiopia
Source: Parasit Vectors. 2025 Jun 19;18:228. doi: 10.1186/s13071-025-06855-3 (PMC12177963; doi:10.1186/s13071-025-06855-3)
Supplement: Supplementary file 1 — Additional file 1: Table S1. Individual data of the cat population distributed in the four investigated sites [file 13071_2025_6855_MOESM1_ESM.docx]

Additional File 1 – Table S1: Individual data of the cat population distributed in the four investigated sites.

| **Animal’s data** | | **Arba Minch town**  **n (%)** | **Arba Minch zuria district**  **n (%)** | **Gerese district**  **n (%)** | **Chencha town n (%)** | **Total**  **n (%)** |
| --- | --- | --- | --- | --- | --- | --- |
| Tested |  | **40** | **32** | **31** | **6** | **109** |
| Sex | Male | 10 (25.0) | 16 (50.0) | 11 (35.5) | 6 (100) | 43 (39.4) |
|  | Female | 30 (75.0) | 16 (50.0) | 20 (64.5) | 0 (0) | 66 (60.6) |
| Age class | Adult (>6m) | 30 (75.0) | 24 (75.0) | 23 (74.2) | 6 (100) | 83 (76.1) |
|  | Young (≤6m) | 10 (25.0) | 8 (25.0) | 8 (25.8) | 0 (0) | 26 (23.9) |
| Lifestyle | Indoor | 34 (85.0) | 24 (75.0) | 28 (90.3) | 3 (50.0) | 89 (81.7) |
|  | Mixed | 6 (15.0) | 5 (15.6) | 2 (6.5) | 3 (50.0) | 16 (14.7) |
|  | Outdoor | 0 (0) | 3 (9.4) | 1 (3.2) | 0 (0) | 4 (3.7) |
| Agroecology | Lowland | 40 (100) | 11 (34.4) | 0 (0) | 0 (0) | 51 (46.8) |
|  | Midland | 0 (0) | 21 (65.6) | 0 (0) | 0 (0) | 21 (19.3) |
|  | Highland | 0 (0) | 0 (0) | 31 (100) | 6 (100) | 37 (33.9) |
| Flea infestation | Presence | 11 (27.5) | 6 (18.8) | 7 (22.6) | 0 (0) | 24 (22.0) |
|  | Absent | 29 (72.5) | 26 (81.3) | 24 (77.4) | 6 (100) | 85 (78.0) |
