# Supplementary material for: Vector-borne pathogens in cats and associated fleas in southern Ethiopia
Source: Parasit Vectors. 2025 Jun 19;18:228. doi: 10.1186/s13071-025-06855-3 (PMC12177963; doi:10.1186/s13071-025-06855-3)
Supplement: Supplementary file 2 — Additional file 2: Table S2. ID numbers of the sequences obtained from positive samples and deposited in GenBank [file 13071_2025_6855_MOESM2_ESM.docx]

Additional File 2 – Table S2: Genbank accession numbers of the sequences obtained from positive sample.

| **Origin** | **N samples** | **Pathogen species** | **gene** | **N sequences** | **GenBank Accession Numbers** |
| --- | --- | --- | --- | --- | --- |
| Cat blood sample | 33 | *Hepatozoon felis* | *18S* - 373bp | 33 | PP542601 - PP542633 |
|  | 6 | *Hepatozoon luiperdjie* | *18S* - 373bp | 6 | PP542639 – PP542644 |
|  |  |  | *18S* - 800bp | 2 | PQ867486 –PQ867487 |
|  | 1 | *Hepatozoon canis* | *18S* - 373bp | 1 | PP542647 |
|  | 1 | *Babesia leo* | *18S* - 373bp | 1 | PP542645 |
|  |  |  | *18S* - 800bp | 1 | PQ867483 |
|  | 1 | *Rickettsia felis* | *gltA* | 1 | PP886884 |
|  |  |  | *ompB* | 1 | PQ899608 |
|  | 5 | *Rickettsia* spp*.* | *gltA* | 5 | PP886885, PQ899609 - PQ899612 |
| Flea  (*C. felis*) | 20 | *Rickettsia asembonensis* | *gltA* | 20 | PP886886 - PP886899, PP886903 - PP886905, PP886943 – PP886945 |
|  |  |  | *ompB* | 18 | PQ899572 – PQ899583, PQ899587 – PQ899592 |
|  |  |  | *htrA* | 4 | PQ899596 – PQ899599 |
|  | 1 | *Rickettsia felis* | *gltA* | 1 | PP886901 |
|  |  |  | *ompB* | 1 | PQ899585 |
|  | 2 | *Rickettsia* spp*.* | *gltA* | 2 | PP886900, PP886902 |
|  |  |  | *ompB* | 2 | PQ899584, PQ899586 |
| Flea  (*E. gallinacea*) | 1 | *Hepatozoon felis* | *18S* - 373bp | 1 | PP542634 |
|  | 1 | *Rickettsia asembonensis* | *gltA* | 1 | PP886908 |
|  |  |  | *ompB* | 1 | PQ899593 |
|  | 36 | *Rickettsia* spp*.* | *gltA* | 36 | PP886906, PP886907, PP886909 – PP886943 |
|  |  |  | *ompB* | 2 | PQ899594, PQ899595 |
|  |  |  | *htrA* | 8 | PQ899600 – PQ899607 |

*Bartonella* identification numbers are not available because the length of ITS nucleotide sequences was less than 150 pb as required by GenBank.
