# Supplementary figures and images for: Vector-borne pathogens in cats and associated fleas in southern Ethiopia
Source: Parasit Vectors. 2025 Jun 19;18:228. doi: 10.1186/s13071-025-06855-3 (PMC12177963; doi:10.1186/s13071-025-06855-3)

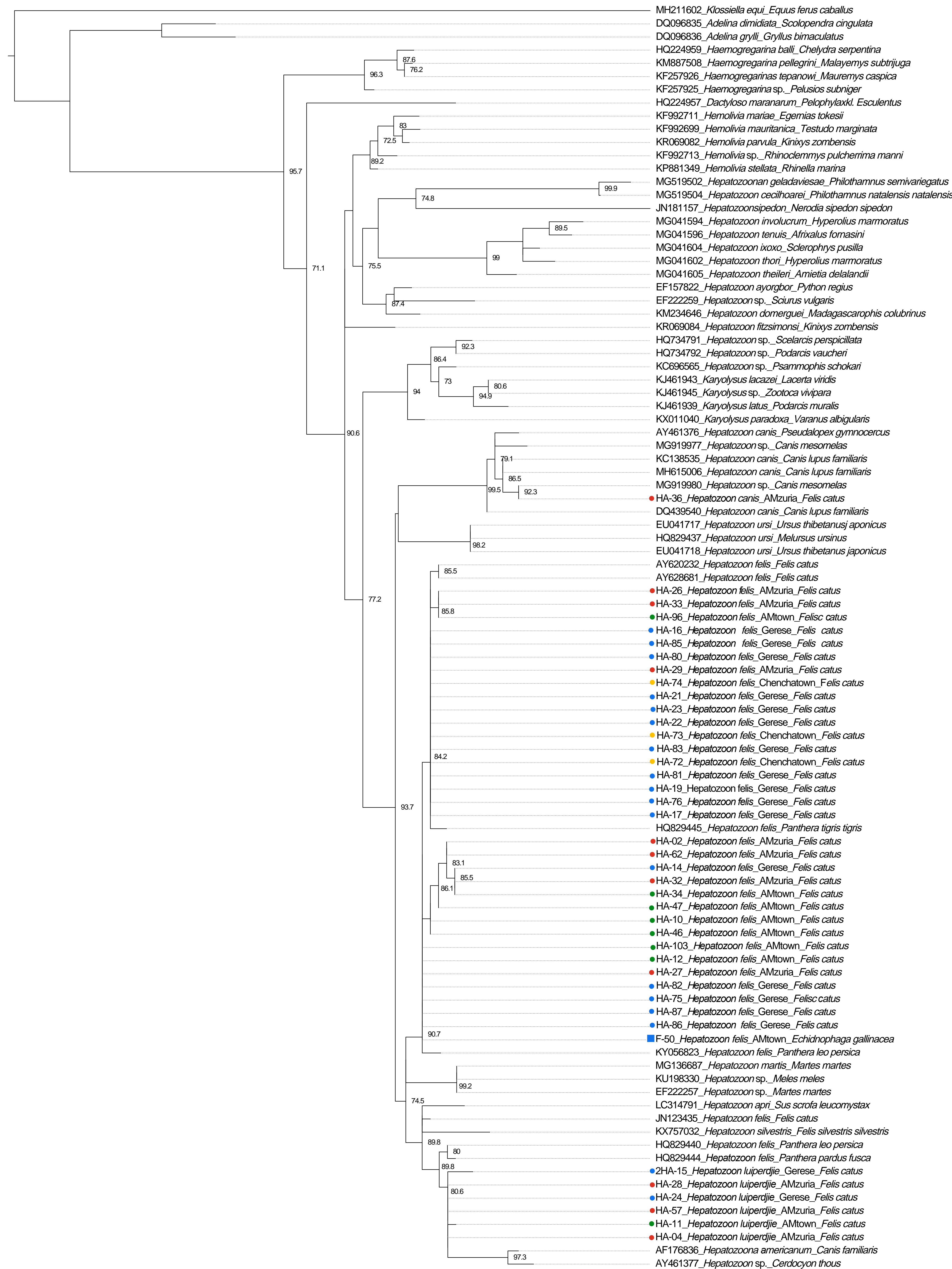

Supplement: Supplementary file 3 — Additional file 3: Fig S1. Maximum likelihood phylogenetic tree of the obtained Hepatozoon sequences plus a set of reference ones. Bootstrap support values higher than 70% have been reported near the corresponding nodes. Sequences obtained from cats have been marked with a filled circle, while the one obtained from Echidnophaga gallinacea has been marked with a filled square. Collection areas have been color-coded: Arba Minch town in green, Gerese district in blue, Arba Minch Zuria district in red, and Chencha town in yellow [file 13071_2025_6855_MOESM3_ESM.pdf]

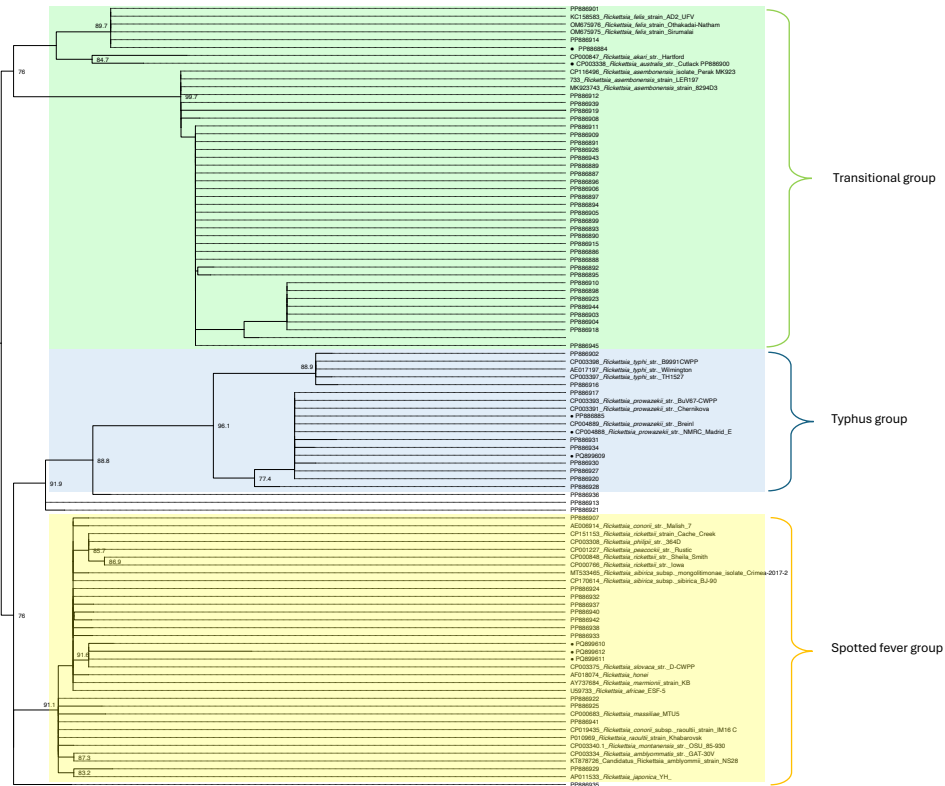

Supplement: Supplementary file 4 — Additional file 4: Fig S2. Maximum likelihood phylogenetic tree of the obtained Rickettsia spp. gltA gene portion sequences obtained in the present study, plus a set of reference ones. Sequences obtained from cats have been marked with a filled circle. Branch support is reported near the corresponding node; only values higher than 70 have been maintained for clarity reasons. The use of boxes with colored background highlights the clustering within the spotted fever group (light yellow), typhus group (light blue), and transitional group (light green). Four sequences (i.e., PP886936, PP886913, PP886921, PP886935) are poorly related to the selected references and therefore are not included in the colored areas. [file 13071_2025_6855_MOESM4_ESM.pdf]
